# Structural and functional studies of HIV-1 pre-integration complexes

**DOI:** 10.1186/1471-2334-14-S2-O9

**Published:** 2014-05-23

**Authors:** Marc Ruff, Nicolas Levy, Sylvia Eiler, Karine Pradeau, Corinne Crucifix, Aurélie Schaetzel, Robert Drillien, Vincent Parissi, Stéphane Emiliani, Yves Mely, Patrick Schultz

**Affiliations:** 1 Institute of Genetics and Molecular and Cellular Biology, Illkirch, France; 2 University of Bordeaux, Laboratory of basic microbiology and pathogenicity, Bordeaux, France; 3 Cochin Institute, Paris Descartes University, Paris, France; 4 Faculty of Pharmacy, Laboratory of biophotonics and pharmacology, Illkirch, France

## 

HIV-1 integrase (IN) is a key component of the pre-integration complex (PIC) and is involved in several steps of retrovirus replication. IN cannot perform these functions on its own and need to recruit host cell proteins to efficiently carry out the different processes. Retroviral INs are flexible proteins showing high inter-domain flexibility. This intrinsic flexibility accounts for IN ability to interact with multiple partners, which in turn chaperones PIC formation and its multiple functions. Biochemical and structural studies have long been hampered due to the PIC dynamics and intrinsic flexibility. We demonstrated that the low solubility and inter-domain flexibility can be circumvented by forming stable and specific complexes with DNA or protein co-factors and by post-translational modifications.

Hundreds of milligrams of stable complexes are needed for in vitro functional and structural studies. For this, we develop new technologies for high molecular weight transient complexes production as well as for functional and structural analysis (mammalian cell system for protein production, in vitro functional analysis and complexes characterization). Structures have been solved by Cryo-EM and X-ray crystallography.

We reconstruct in vitro stable and soluble complexes around IN. We solved the cryo-EM structures of the IN/LEDGF/DNA (Michel et al. 2009) and IN/LEDGF/INI1/DNA (Maillot et al. 2013) complexes. Structures together with functional assays gave important hints on the functional role of LEDGF and INI1. Other sub-complexes of the PIC have been characterized. One of them is the IN/transportin-SR2/VBP1 complex (structure in progress). Our results suggest that the function of INI1 (core protein of the swi/snf chromatin remodeling complex) in HIV-1 replication is to stabilize the PIC in the host cell, by maintaining integrase in a stable constrained conformation which prevents non-specific interactions and auto integration on the route to its integration site within nucleosomes, while LEDGF (a transcriptional co-activator) organizes and stabilizes an active integrase tetramer suitable for specific vDNA integration.

